# Distinct migratory and non-migratory ecotypes of an endemic New Zealand eleotrid (*Gobiomorphus cotidianus*) – implications for incipient speciation in island freshwater fish species

**DOI:** 10.1186/1471-2148-8-49

**Published:** 2008-02-14

**Authors:** Christian Michel, Brendan J Hicks, Kai N Stölting, Andrew C Clarke, Mark I Stevens, Ray Tana, Axel Meyer, Michael R van den Heuvel

**Affiliations:** 1 Limnological Institute, University of Konstanz, Konstanz, Germany; 2 Centre for Biodiversity and Ecology Research, Department of Biological Sciences, University of Waikato, Hamilton, New Zealand; 3 Zoological Museum, University of Zürich, Zürich, Switzerland; 4 Allan Wilson Centre for Molecular Ecology and Evolution, Massey University, Palmerston North, New Zealand; 5 Institute of Molecular BioSciences, Massey University, Palmerston North, New Zealand; 6 School of Biological Sciences, Flinders University, Adelaide, SA, Australia; 7 Evolutionary Biology, University of Konstanz, Konstanz, Germany; 8 Canadian Rivers Institute, University of Prince Edward Island, Prince Edward Island, Canada

## Abstract

**Background:**

Many postglacial lakes contain fish species with distinct ecomorphs. Similar evolutionary scenarios might be acting on evolutionarily young fish communities in lakes of remote islands. One process that drives diversification in island freshwater fish species is the colonization of depauperate freshwater environments by diadromous (migratory) taxa, which secondarily lose their migratory behaviour. The loss of migration limits dispersal and gene flow between distant populations, and, therefore, is expected to facilitate local morphological and genetic differentiation. To date, most studies have focused on interspecific relationships among migratory species and their non-migratory sister taxa. We hypothesize that the loss of migration facilitates intraspecific morphological, behavioural, and genetic differentiation between migratory and non-migratory populations of facultatively diadromous taxa, and, hence, incipient speciation of island freshwater fish species.

**Results:**

Microchemical analyses of otolith isotopes (^88^Sr, ^137^Ba and ^43^Ca) differentiated migratory and non-migratory stocks of the New Zealand endemic *Gobiomorphus cotidianus *McDowall (Eleotridae). Samples were taken from two rivers, one lake and two geographically-separated outgroup locations. Meristic analyses of oculoscapular lateral line canals documented a gradual reduction of these structures in the non-migratory populations. Amplified fragment length polymorphism (AFLP) fingerprints revealed considerable genetic isolation between migratory and non-migratory populations. Temporal differences in reproductive timing (migratory = winter spawners, non-migratory = summer spawners; as inferred from gonadosomatic indices) provide a prezygotic reproductive isolation mechanism between the two ecotypes.

**Conclusion:**

This study provides a holistic look at the role of diadromy in incipient speciation of island freshwater fish species. All four analytical approaches (otolith microchemistry, morphology, spawning timing, population genetics) yield congruent results, and provide clear and independent evidence for the existence of distinct migratory and non-migratory ecotypes within a river in a geographically confined range. The morphological changes within the non-migratory populations parallel interspecific patterns observed in all non-migratory New Zealand endemic *Gobiomorphus *species and other derived gobiid taxa, a pattern suggesting parallel evolution. This study indicates, for the first time, that distinct ecotypes of island freshwater fish species may be formed as a consequence of loss of migration and subsequent diversification. Therefore, if reproductive isolation persists, these processes may provide a mechanism to facilitate speciation.

## Background

Teleost fish exhibit astonishing examples of adaptive evolution, such as observed in the African cichlids [1], the Neotropical Midas cichlids [2], and the limnetic and benthic stickleback morphs [3]. In general, the colonization of new environments allows rapid diversification [4] as a by-product of adaptation to divergent selection regimes [5], and can finally lead to reproductive isolation [6]. Well known examples of this process in the Northern Hemisphere include the formation of distinct ecotypes in many species pairs of postglacial freshwater fish [7]. Evidence for ecological speciation in these species pairs includes the rapid evolution of reproductive isolation (e.g., separate breeding times, paucity of morphological hybrids) and the parallel evolution of inherited morphological differences that indicate specialization for different niches [7]. Ecotype divergence in the Northern Hemisphere is apparent in numerous species pairs of freshwater fish [8-12], and also includes some anadromous (adults migrating from salt water to spawn in fresh water) and freshwater resident pairs [13,14]. In contrast, published examples of ecotype divergence in the Southern Hemisphere are sparse [11].

Evolutionary processes comparable to those observed in the Northern Hemisphere postglacial lakes have also likely occurred in oceanic island groups (e.g., Hawai'i, Falkland Islands, Tasmania and the Marquesas Islands), where recently formed lakes with a depauperate freshwater fauna were secondarily colonized by diadromous (migratory between salt and freshwater) fish species that then lost their migratory behaviour [15-17]. Compared to the Northern Hemisphere, in the Southern Hemisphere anadromy is much less common [16], and the majority of diadromous species are amphidromous – a special form of diadromy in which only larvae drift to sea and early juveniles (15–50 mm) return to freshwater [18,19]. The adaptive significance of amphidromy is the maintenance of dispersal between isolated, tectonically active island land masses, thereby maintaining gene flow among geographically distant populations [18,20-23]. Accordingly, the loss of migration in amphidromous species leads to geographic isolation and is believed to have initiated genetic and morphological diversification in many taxa [16,24,25]. Well known examples of this process are the freshwater radiations of galaxiid fishes, the diversifications of which have likely been driven by landlocking [26-30]. Consequently, extensive genetic population structuring is observed in several non-migratory species [31,32], including the New Zealand endemic *Gobiomorphus breviceps *[33]. Of seven New Zealand endemic *Gobiomorphus *species, three are obligatorily freshwater resident, three are obligatorily amphidromous [34], while only the widespread and facultatively amphidromous [35]*Gobiomorphus cotidianus *McDowall readily establishes non-migratory populations [34]. One of the obligatorily freshwater resident species (*G. alpinus *[36]) arose within the last 18,000 years [37], and is closely related to *G. cotidianus *[36,37]. All New Zealand representatives of the genus *Gobiomorphus *represent a radiation within the basal Gobioidei [38]. This island *Gobiomorphus *complex forms a monophyletic group [38], whose ancestor most likely arrived by means of oceanic dispersal ([39], M.I. Stevens & B.J. Hicks, unpublished cytochrome *b *data). The extensive genetic structuring observed in the non-migratory *G. breviceps *[33], as well as the non-migratory and recently evolved *G. alpinus *clearly suggest that the loss of the marine larval life stage facilitates diversification in the New Zealand *Gobiomorphus *complex.

In gobiids, the structure of the peripheral lateral line canals is an important taxonomic character [40]. In addition, the morphological patterns of these canals can be correlated with particular hydrodynamic stimuli that have direct fitness consequences for fishes (e.g., during rheotaxis, prey detection or predator avoidance) [41-44]. Canal reduction is thought to be an adaptation to distinct microhabitats with slow-flowing water conditions [45], and the congruence between genetic structure and geographic distribution of oculoscapular canal morphotypes in the tidewater goby *Eucyclogobius newberry *suggests that these variations can be partly inherited [46]. *Gobiomorphus cotidianus *with predominantly superficial lateral line neuromasts exhibit better detection of moving objects in the absence of background flow [47]. The structure of the oculoscapular lateral line canals in *G. cotidianus *is highly variable, including complete absence in some lake populations [34]. Furthermore, all obligatorily amphidromous New Zealand *Gobiomorphus *species have fully developed oculoscapular canals, while these canals are completely absent in all obligatorily non-migratory species [34], a pattern suggesting parallel evolution.

**Figure 1:**
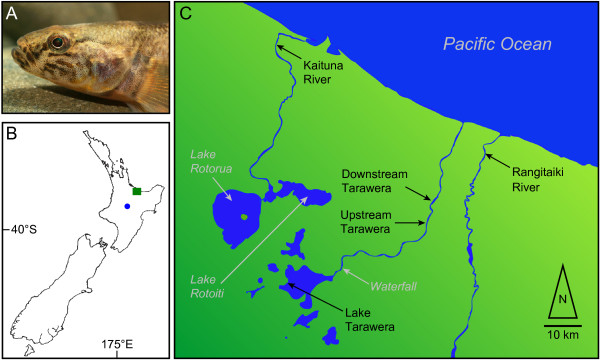
**Study area and sample locations for *Gobiomorphus cotidianus***. A: Lateral view on the head of a male *Gobiomorphus cotidianus *(^© ^Angus McIntosh, Natural Sciences Image Library, New Zealand). B: Location of the study area in New Zealand (green square) and location of Lake Taupo (blue dot). C: Study area with sample locations (black) for *G. cotidianus *and geographic locations (grey/italic) as mentioned in the text.

The North Island of New Zealand contains numerous recently-formed lakes [48] that have a diverse history of catastrophic events (e.g., volcanism, glaciations, and sea level changes) that have allowed subsequent colonisations by amphidromous taxa [49]. One of these lakes is Lake Tarawera (Figure 1), a large (39 km^2^) and deep (90 m) oligotrophic lake located in the geologically highly active central Okataina dome. Lake Tarawera was formed about 10,000 years ago [50]. It drains into the Tarawera River, which is characterized by fast-flowing upper river reaches, including a waterfall (Tarawera Falls, height 65 m, ~2 km from the lake outlet) that represents a significant upstream dispersal barrier for fish into the lake. However, dispersal out of the lake is possible, and major pulses of downstream transport of fish caused by the collapse of lava flows have likely occurred [51]. About 35 km downstream of the waterfall the river enters an extended area of flat land before reaching the Pacific Ocean. No physical barriers limit downstream dispersal of fish within the Tarawera River, whereas upstream dispersal may be limited [52]. The Kaituna River, which originates in the nearby Lake Rotoiti, drains into a coastal area before also reaching the Pacific Ocean. The river mouths of the Tarawera and Kaituna rivers are separated by 50 km of coastline (Figure 1). The Tarawera and Kaituna river systems share no freshwater connections. Like Lake Tarawera, Lake Rotoiti was also affected by volcanic activities. The Rangitaiki River originates from a separate geographic area and drains into the sea about 12 km away from the Tarawera River (Figure 1). Prior to modifications for flood protection (*ca *1900) the Tarawera and Rangitaiki rivers likely shared freshwater connections under flood conditions. Following several volcanic eruptions (AD 186 and 1886) that eliminated most freshwater fauna from Lake Tarawera and the surrounding lakes [49], forage fish for trout were introduced into lakes Rotorua and Rotoiti (Figure 1) and from there into Lake Tarawera (around 1900; [53]). These fish likely included *G. cotidianus *specimens obtained from the Waikato River, a river system originating in the central North Island lake of Lake Taupo (616 km^2^; Figure 1).

The loss of the migratory life stage is a key process driving speciation in island freshwater fish species [54]. We hypothesize that the loss of the migratory life stage in facultatively amphidromous taxa facilitates intraspecific morphological, behavioural, and genetic differentiation between migratory and non-migratory ecotypes, thereby providing a mechanism for incipient speciation. To date, most studies have focused on interspecific relationships among diadromous species and their non-migratory sister taxa, while little attention has been given to incipient speciation occurring as a result of intraspecific evolutionary processes. Accordingly, the facultatively amphidromous *G. cotidianus *offers an excellent opportunity to study morphological, behavioural, and genetic diversification between amphidromous and freshwater resident (i.e. non-migratory) populations.

**Figure 2:**
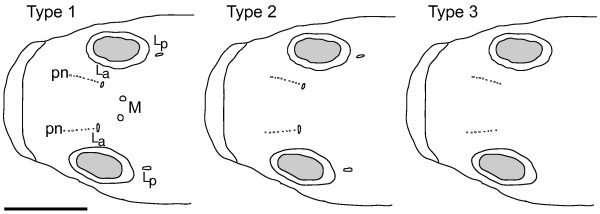
**Schematic dorsal view of the oculoscapular canal section of the defined canal morphotypes**. The canal structures are evident by presence of pores at their extremities. Median pores (M), lateral pores (L_a _= anterior, L_p _= posterior lateral pore) and primary neuromasts (pn). Scale bar = 10 mm.

To test for morphological, reproductive, and genetic diversification between migratory and non-migratory stocks, we collected *G. cotidianus *from one lake and two river sample sites in the Tarawera system (Figure 1). Outgroup samples from the nearby Kaituna and Rangitaiki rivers (Figure 1) were included to examine possible differences between river systems (Table 1). Otolith microchemical analyses of ^88^Sr, ^137^Ba and ^43^Ca isotopes were used to distinguish migratory and non-migratory stocks. The analysis was complemented with analyses of ^137^Ba/^43^Ca ratios, as changing levels of this isotope across the otolith indicate diadromy [55]. To test for possible morphological differences between migratory types, the otolith data were contrasted with the distribution of oculoscapular canal morphotypes (as determined from the pore openings of the canals; Figure 2). Previous work suggested the presence of distinct summer and winter spawning populations in the Tarawera River [52]. Thus, we included data on the gonadal development (gonadosomatic indices) to test for temporal reproductive isolation among migratory and non-migratory stocks. All analyses were contrasted with the genetic structure as inferred by Amplified Fragment Length Polymorphisms (AFLPs; [56]), a selectively-neutral, high resolution marker system that can generate a high number of markers distributed genome-wide [57,58]. AFLPs are capable of resolving recent evolutionary splits [59-61], such as expected between different ecotypes. This multidisciplinary approach was applied to reveal patterns of diversification occurring in the New Zealand *Gobiomorphus *complex as a consequence of loss of migration, and, therefore, the role of this process in driving speciation of island freshwater fish species.

**Table 1:** Sample details and descriptive parameters for meristic, otolith, reproduction and genetic analyses.

Site	Meristics	Otoliths		Reproduction			Genetic analyses (AFLPs)		
				
	*N*_M_	*N*_O_	%_ND_	*N*_R_	%_S_	Stat.	*N*_G_	%_P_	*H*_SW_
LT	30	6	100%	9	100%	A	19	62%	105
UT	16	14	100%	46	87%	A	14	52%	96
DT	29	18	33%	18	6%	B	13	64%	118
RR	18	11	27%	17	0%	B	4	43%	94
KR	19	11	100%	14	64%	A	5	33%	73

LT = Lake Tarawera, UT = upstream Tarawera, DT = downstream Tarawera, RR = Rangitaiki River, KR = Kaituna River. *N*_M _= Samples included in meristic analyses, N_O _= Samples included in otolith analyses, %_ND _= Percentage of non-diadromous fish, *N*_R _= Female samples included in reproduction-type comparisons, %_S _= Percentage of summer spawning females, Stat. = Significant differences (*P *< 0.05) in spawning type composition, shared letters indicate no significant difference at *P *< 0.05. *N*_G _= Samples included in genetic analyses, %_P _= Percentage polymorphic fragments (from 732 total fragments), *H*_SW _= Shannon-Wiener Diversity index.

## Results

### Meristic analysis

All fish included here were sexually mature (> 60 mm; [34]), so their canal formation was complete. No differences in canal formation between sexes were observed. In all individuals, the anterior section of the lateral oculoscapular canals was reduced, exposing a row of primary neuromasts that terminate at pore L_a _(Figure 2). No specimens were found with median pores present and lateral pores lacking. Hence, the median pores were always reduced first, followed by a gradual reduction of the lateral canals, spanning between pores L_a _and L_p _(Figure 2) from anterior to posterior. Canal development was most pronounced (Type 1; Figure 2) in samples from the downstream Tarawera (83%) and the Rangitaiki River (67%) samples (Table 2). Only the individuals with paired median pores absent but lateral pores present (Type 2; Figure 2) were present in all sample locations, with highest proportions (Table 2) in upstream Tarawera (81%) and the Kaituna River (61%). Nearly all individuals in Lake Tarawera and upstream Tarawera exhibited degraded oculoscapular canals (Type 2; 33% and 81% respectively) or had lost all canals (Type 3; 60% and 19% respectively). The highest mean proportion of fish without canals was found in Lake Tarawera (60%). Morphotype composition differed significantly among sample locations (Kruskal-Wallis ANOVA: *N *= 112, d.f. = 4, *H *= 58.5, *P *< 0.01). Results of pair-wise *post hoc *comparisons are given in Table 2. Asymmetrically reduced lateral pores (i.e., present on one side only) were only observed in the Lake Tarawera and upstream Tarawera sites (data not shown).

**Table 2:** Morphotypes in each sample site.

Site	***N***_M_	Type 1	Type 2	Type 3	Stat.
LT	30	7%	33%	60%	A
UT	16	0%	81%	19%	AB
DT	29	83%	13%	3%	C
RR	18	67%	33%	0%	C
KR	19	39%	61%	0%	BC

LT = Lake Tarawera, UT = upstream Tarawera, DT = downstream Tarawera, RR = Rangitaiki River, KR = Kaituna River. *N*_M _= Number of individuals per sample site included in meristic analyses. Type 1, 2 and 3 = proportion of the respective morphotype (Figure 2) in sample site. Stat. = Significant differences in morphotype compositions between sample sites, shared letters indicate no significant difference at *P *< 0.05.

### Otolith isotope profiles

**Figure 3:**
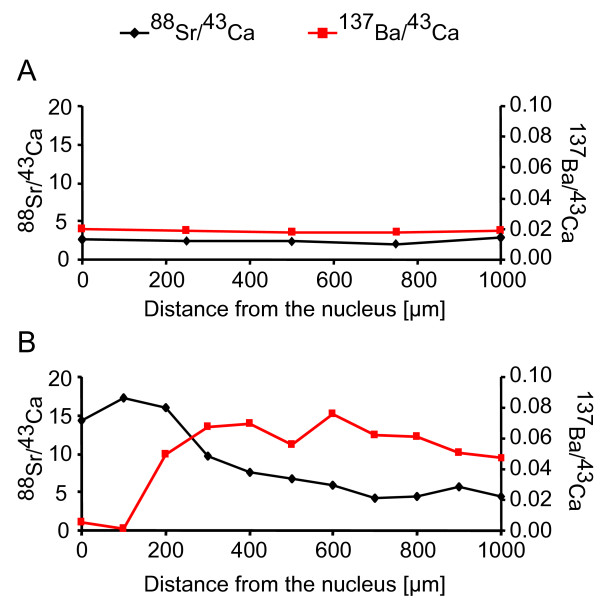
**Typical patterns of ^88^Sr and ^137^Ba counts normalised to ^43^Ca in otolith cross sections from the nucleus to the edge**. A: non-diadromous profile (category A) in a female *Gobiomorphus cotidianus *(92 mm) from the Kaituna River. B: diadromous profile (category B) in a male *G. cotidianus *(96 mm) from the downstream Tarawera River.

Elevated ^88^Sr/^43^Ca ratios in fish otoliths are widely accepted as proof of occupation of marine habitats [55,62-65]. Similarly, in amphidromous *G. cotidianus*, elevated ^88^Sr/^43^Ca ratios in the otoliths nucleus indicate a marine larval life stage [35]. Obligatorily amphidromous *G. gobioides *sampled in the Tarawera River were used to establish reference isotope profiles for amphidromous individuals, allowing migratory and non-migratory *G. cotidianus *to be distinguished. Thus, distinct differences among individual isotope profiles permitted all samples to be grouped into one of two distinct categories:

(A) Non-diadromous individuals have low ^88^Sr/^43^Ca ratios in the nucleus (< 2.5; Figure 3A) with a small range (< 1.5). Their freshwater residency was also supported by a constant level of normalised ^137^Ba/^43^Ca from nucleus to edge.

(B) Diadromous individuals have higher ^88^Sr/^43^Ca ratios in the nucleus (> 4.5) and a larger range between the nucleus and the edge (> 2.0; Figure 3B), illustrating a marine or estuarine larval life stage. In these profiles, the larval migration was also reflected in a characteristic signature of decreasing ^88^Sr/^43^Ca ratios and increasing ^137^Ba/^43^Ca ratios from the nucleus to the otolith edge (Figure 3B).

**Figure 4:**
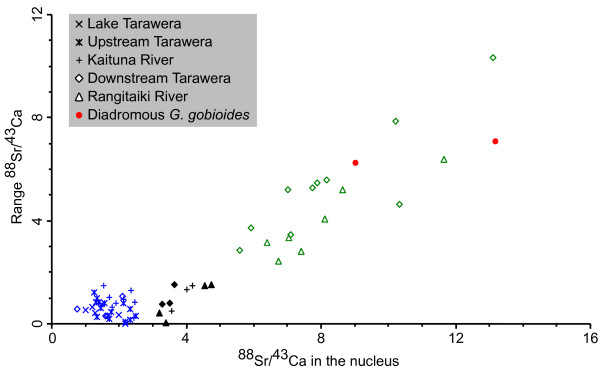
**Nucleus counts against range of normalised ^88^Sr/^43^Ca in *G. cotidianus *and two obligatorily diadromous *G. gobioides *(red solid dots) included in this study**. Colours refer to migratory types: blue = non-diadromous, green = diadromous. Black solid symbols were not confidently attributable to either category based on nucleus vs range counts.

The ^88^Sr/^43^Ca ratios in the otolith nucleus were lower in non-diadromous fish (mean = 2.2, *N *= 40) than in diadromous individuals (mean = 11.4, *N *= 20; ANOVA *F*_1.58 _= 139, *P *< 0.001). Comparisons of the individual nucleus isotope patterns can be seen in Figure 4. A few individuals could not be confidently allocated to either category based on the descriptive characters used in the scatter plot (Figure 4; solid symbols). However, all amphidromous *G. cotidianus *exhibited a characteristic increase in ^137^Ba/^43^Ca from nucleus to the edge, while all freshwater resident individuals showed a constant level of ^137^Ba/^43^Ca across the otolith (Figure 3). Therefore, the relative differences in individual isotope profiles were used to allocate these individuals to one of the two categories. All Lake Tarawera and upstream Tarawera specimens cluster together (Figure 4) and possess non-diadromous isotope profiles, confirming a complete freshwater life history (Table 1). Similarly, all Kaituna River samples were non-diadromous, as no otolith from this site showed evidence of a marine larval life stage (Table 1). In the downstream Tarawera site, 66% of specimens were diadromous, while from the Rangitaiki River, 73% were diadromous (Table 1).

### Distribution of spawning types

The proportions of female spawning types are given in Table 1. Significant differences in spawning type proportions among sample sites are observable (Kruskal-Wallis ANOVA: *N *= 104, d.f. = 4, *H *= 52.3, *P *< 0.01). Pair-wise *post hoc *comparisons are given in Table 1. Specimens in Lake Tarawera, upstream Tarawera and Kaituna River spawned predominantly in summer (Table 1). Gonads of the Lake Tarawera females captured in September (spring) were translucent and homogenous in colour with no sign of recent spawning. This differed significantly (Table 1) to the downstream Tarawera and Rangitaiki River sites, which were dominated by winter spawners. Their gonads were consistently in a refractory period of reproductive development during late spring or summer, supporting their status as winter spawners.

### Genetic structure

**Figure 5:**
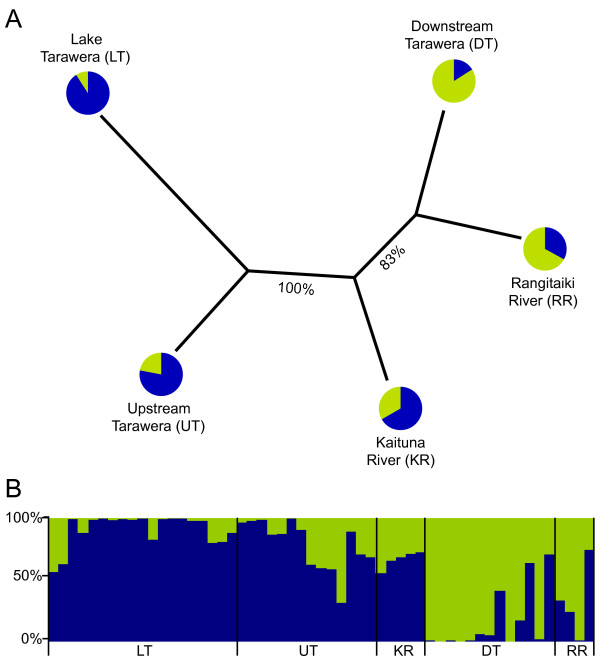
**Analyses of genetic structure among sample locations as inferred from AFLP fingerprints**. A: Population dendrogram. Numbers on branches are percent bootstrap values out of 1,000,000 pseudo-replicates: Pie diagrams at branch ends illustrate mean proportions of inferred genotypes in the respective sample sites. B: Detailed STRUCTURE bar plot illustrating genotypic composition. Colours are inferred green (light shading) and blue (dark shading) genotypes. X-axis: each vertical bar represents one individual. Y-axis: proportion of genotypes. Labels refer to sample sites as given in A.

A large number of AFLP fragments were scored (732), 92% of which were polymorphic. The number of fragments scored and the degree of polymorphism is similar to other studies utilizing AFLPs to distinguish recent evolutionary splits [2,61,66,67]. Both *F*_ST _and θ_B _consistently indicated significant genetic differentiation between most sample sites, except between downstream Tarawera and the Rangitaiki River, and between the Kaituna River and the Rangitaiki River (Table 3). Within the Tarawera system, genetic differentiation between the upstream Tarawera River and Lake Tarawera sites (*F*_ST _= 0.05, *P *< 0.01) was approximately threefold smaller than between the upstream and downstream Tarawera sites (*F*_ST _= 0.13, *P *< 0.01). The population dendrogram (Figure 5A) shows two distinct clusters (LT, UT and DT, RR), with the Kaituna River samples in an intermediate position. In the STRUCTURE analyses, multiple runs with the same K lead to virtually the same result. The method of Evanno et al. [68] revealed a global maximum of ΔK for two clusters (K = 2; ΔK = 235.7), therefore we present the bar plot for two clusters (Figure 5B). The bar plot clearly indicates a high genetic similarity between the Lake Tarawera and the upstream Tarawera population (shared blue genotype) as well as between the downstream Tarawera and the Rangitaiki River sample site (shared green genotype). The Kaituna River is dominated by the blue genotype. The inferred structure agrees with the dendrogram (Figure 5A).

**Table 3:** Genetic differentiation (*F*_ST _and θ_B_) between sample sites.

	LT	UT	DT	RR	KR
LT		0.04*	0.19*	0.11*	0.10*
UT	0.05**		0.12*	0.11*	0.14*
DT	0.20**	0.13**		0.01^ns^	0.10*
RR	0.13**	0.12**	0.02^ns^		0.02^ns^
KR	0.13**	0.17**	0.15**	0.05^ns^	

LT = Lake Tarawera, UT = upstream Tarawera, DT = downstream Tarawera, RR = Rangitaiki River, KR = Kaituna River. Below diagonal *F*_ST_, above diagonal θ_B_, significantly distinct at *P < 0.05, **P < 0.01, ^ns ^= no significant difference.

### Comparisons of the different analyses

**Figure 6:**
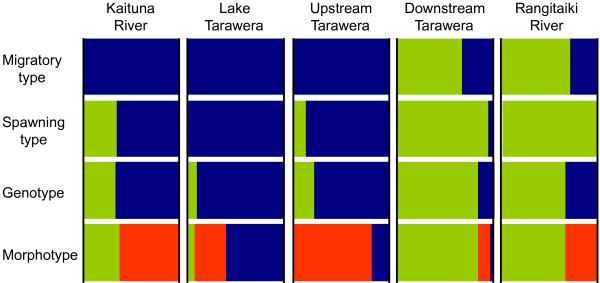
**Summarized comparison of otolith, meristic, reproductive and genetic analyses**. Sample locations are indicated above figure, with each characterized by four vertically arranged bar plots. Horizontal arrangements: Migratory type: light green = diadromous, dark blue = non-diadromous. Spawning type: light green = winter spawner, dark blue = summer spawner. Genotype: mean proportions of respective genotype (light green and dark blue) in population, colours refer to genotype as inferred by STRUCTURE (see also Figure 5). Morphotype: green (light) = Type 1, orange = Type 2, blue (dark) = Type 3.

The results of all analyses are graphically summarized in Figure 6. Populations with dominance of the migratory ecotype (downstream Tarawera, Rangitaiki River) are characterized by a high proportion of the green genotype and a dominance of fish with full canals that predominantly spawn in winter. In contrast, the populations dominated by non-migratory fish (Lake Tarawera, upstream Tarawera, Kaituna River) exhibited a high proportion of the blue genotype and individuals with reduced canals (Type 2 and Type 3) that predominantly spawn in summer.

## Discussion

### Canal reduction in non-migratory populations

The clear differences in migratory behaviour and canal morphotype proportions between the downstream and upstream Tarawera River sites – which are separated by less than 10 km – is remarkable. The absence of migratory fish in upstream Tarawera could be explained by limited upstream migration through the paper mill effluent outfalls [52], or by inherited behavioural patterns. The substantial variations observed in the oculoscapular canal system are clearly not evenly distributed across the sample sites. Notably, fish without oculoscapular canals (Type 3) are largely absent from the migratory downstream Tarawera and Rangitaiki River sites, whereas specimens with fully developed canals (Type 1) are nearly absent from the lake-locked Lake Tarawera, and the non-migratory upstream Tarawera and Kaituna River sites. In *G. cotidianus *without canals, detection of moving objects is most sensitive in the absence of background flow, with sensitivity substantially decreasing as flow velocity increases [47]. Furthermore, reduced oculoscapular lateral line canals can be found in various unrelated fish species that occupy low noise environments [43,45,69]. The pattern of canal reduction in the non-migratory populations is consistent with canal reduction in many lake populations of *G. cotidianus*, and closely resembles the absence of oculoscapular canals in all freshwater resident New Zealand endemic *Gobiomorphus *species [34], a pattern suggesting parallel evolution. Similar patterns of parallel evolution can be observed in the Northern Hemisphere limnetic and benthic forms of *Gasterosteus aculeatus *[3], and in the trophic morphs of *Salvelinus alpinus *[70], with both forms of each species having evolved repeatedly in different lakes. Repeated evolution of similar traits in closely related species that are consistent with similar transitions in the environment strongly suggests convergent evolution [71]. Thus, the high proportion of fish with reduced canals in our non-migratory populations is most likely an adaptation to a low-noise environment, and has likely been paralleled by the transition to a non-migratory life cycle.

### Evidence for inherited morphological differences

If the observed canal formation were caused solely by a phenotypic plastic response to the environment then we would expect greatest canal formation in the upstream Tarawera River site, because these fish are exposed to faster flowing water than any other river population examined. However, we observe that the upstream Tarawera population contains the highest proportion of fish with reduced canals among all river populations. Moreover, the Kaituna River samples also exhibit a higher proportion of reduced canals than the downstream Tarawera and the Rangitaiki River populations. Additionally, the majority of the Kaituna River fish spawn in summer and retain a non-migratory behaviour. It is remarkable that the Kaituna River fish possess these non-migratory attributes, given they have open access to the sea. The parallel pattern of reduced canals in the upstream Tarawera and Kaituna River fish could be explained by both these populations originating from non-migratory lake fish that have been washed out from lakes Tarawera and Rotoiti respectively (the *G. cotidianus *in these lakes share a common ancestry due to anthropogenic introductions [53]; see also discussion below). There is a trend, however, that the fish from upstream Tarawera and Kaituna River (both living in a flowing water environment) develop more canals than their lake counterparts, although they do not develop the full set of canals.

The large number of scored AFLP fragments (732) enables us to resolve the genetic structure within and between sample sites (Figure 5), even with relatively small sample sizes from the Kaituna and Rangitaiki rivers. These analyses clearly indicate genetic similarity between the Lake Tarawera, upstream Tarawera and Kaituna River sites (Figure 5, Table 3). In all three sample sites, the genetic similarity is paralleled by a dominance of fish with reduced canals (Figure 6). Of all non-migratory individuals, the Kaituna River samples have been collected closest to the sea. Accordingly, the higher proportion of the green genotype in the non-migratory Kaituna River fish might also indicate ongoing hybridization of non-migratory lake (washed out from Lake Rotoiti) and migratory river ecotypes. This is consistent with the dendrogram results (Figure 5; Kaituna River in an intermediate position) and the genetic distances (Table 3; similar distances between Lake Tarawera-Kaituna, and between Kaituna-downstream Tarawera). This intermediate position of the Kaituna River samples is also reflected in a higher proportion, compared to the upstream Tarawera site, of fish with full canals (Type 1). In contrast, the downstream Tarawera and the Rangitaiki River sites, that are genetically close to each other but genetically distinct from the upstream Tarawera and Lake Tarawera sites (Figures 5; Table 3), are dominated by a high proportion of individuals with full canal development (Type 1) that are amphidromous (Figures 5, 6 and Table 3).

Collectively, the concordance of the canal morphotypes with the genetic structure and the reproductive timing suggests that the observed canal morphology is partly inherited rather than a completely plastic response to the environment. But, as in other gobiids [46], we expect that canal development in *G. cotidianus *is controlled by the interplay of both environmental and genetic factors. Hence, the intermediate pattern of canal formation observed in the upstream Tarawera and the Kaituna River populations may be affected by both a phenotypic plastic response to the ambient river environment, and unidirectional downstream gene flow of non-migratory lake stocks (from lakes Tarawera and Rotoiti respectively) followed by hybridisation with migratory river stocks.

Although we cannot conclude with certainty whether the documented ecotypes are continuing to diverge or are collapsing (such as observed in a *Gasterosteus aculeatus *species pair [72]), we hypothesise that they are more likely to be diverging because of the apparent temporal reproductive isolation of the two ecotypes. Accordingly, incipient speciation may be occurring, particularly in the lake-locked Lake Tarawera population.

### Temporal reproductive isolation

The female reproductive data presented here and in previous work [52] document an approximately six month shift in spawning time between upstream Tarawera (spawning in summer) compared to downstream Tarawera and Rangitaiki rivers (spawning in winter). All female fish from Lake Tarawera exhibited gonadosomatic indices greater than 1.35, accompanied by homogenous and translucent ovaries indicative of the pre-spawning vitellogenic stage. This indicates resource accumulation for spawning during summer. Additionally, the Lake Tarawera samples collected in January showed clear signs of recent spawning, while samples from June 2001 and 2002 did not indicate any gonad development (M.R. van den Heuvel, unpub. data), further supporting their status as summer spawners. This is consistent with summer spawning of other lake populations of *G. cotidianus *[73]. Both Kaituna River and upstream Tarawera populations were collected in January during the peak time of summer spawning of *G. cotidianus *in that region. In contrast, the downstream Tarawera and Rangitaiki River populations spawn in winter. Hence, the pattern of reproductive timing parallels the patterns of canal reduction, migratory behaviour, and the genetic structure. A similar difference in reproductive timing has been found between diadromous and non-diadromous populations of *Galaxias truttaceus *(Galaxiidae) [28]. In that study, the non-diadromous lake-locked population spawns in spring, whereas the diadromous river population spawns in autumn. The authors suggest this shift in spawning time is a precursor to loss of diadromy, as the larvae no longer drift to sea for feeding and may not survive the cold, unproductive lake during winter. The abundance of *G. cotidianus *in the lakes of the North Island of New Zealand is related to lake productivity and therefore food availability [74]. Thus, the observed shift in spawning of our non-migratory populations may be a necessary adaptation to seasonal differences in food availability, but may also provide a prezygotic mechanism to reproductively isolate the non-migratory and migratory populations.

### Evidence for distinct ecotypes

The canal reduction observed in the non-migratory populations of *G. cotidianus *parallels reduced oculoscapular canals in all obligatorily non-diadromous New Zealand *Gobiomorphus *species (all obligatorily diadromous species exhibit fully developed canals, [34]). Thus, the pattern we observe may be the result of equivalent evolutionary processes that have occurred repeatedly during the formation of non-migratory *Gobiomorphus *species in New Zealand. Additional support for this hypothesis can be found in the closely related *G. alpinus*, a recently (less than 18,000 years) evolved sister taxon [36] of *G. cotidianus *that likely originated from a diadromous stock [36,37,75]. The two taxa are indistinguishable based on mtDNA sequences but can be separated using AFLPs (C. Michel & M.I. Stevens, unpub. data), suggesting a recent diversification. Similar to *G. alpinus*, our Lake Tarawera and upstream Tarawera River specimens exhibit reduced counts of rays and spines in the first and second dorsal and anal fin (data not shown), consistent with another pattern of parallel evolution. Within the Gobioidei, the reduction of morphological features (i.e., canals, body size, fin rays, vertebrae) is characteristic of a more derived evolutionary state [76], and the canal reduction observed in our study resembles convergent evolution observed in other derived gobiid [40,46,77-79] and teleost taxa [69,80].

Based on the current data, it is not possible to establish whether the observed morphological and behavioural differences evolved within Lake Tarawera, or in the Lake Taupo/Waikato River system, because it is possible that the non-migratory ecotype originates from the Waikato River and has been introduced into lakes Tarawera and Rotorua/Rotoiti via anthropogenic fish introductions [53]. It is also not yet possible to establish the age of divergence of the two ecotypes. While we cannot rule out that the non-migratory ecotype evolved elsewhere, prior to its introduction, our data clearly support morphologically, reproductively and genetically distinct ecotypes that are derived from distinct migratory and non-migratory stocks.

## Conclusion

Our data are congruent, and provide clear and independent lines of evidence for distinct non-migratory and migratory ecotypes on a geographically small scale along a river. The morphological changes observed in the non-migratory populations closely resemble evolutionary patterns repeatedly observed during formation of freshwater resident *Gobiomorphus *species in New Zealand as well as in other derived gobiid species. These patterns suggest parallel evolution. The present study is, to our knowledge, the first example that clearly suggests that distinct intraspecific ecotypes of an amphidromous fish species (as determined from morphological, reproductive, behavioural, and genetic evidence) may be formed as a consequence of loss of migration and subsequent divergence. Hence, if the reproductive isolation is maintained, these processes could, in the long term, result in the formation of new island freshwater fish species. Future research could focus on several intriguing aspects, including establishing the age of divergence of the ecotypes, exploring the role of landlocking and evolutionary mechanisms (e.g. natural selection, genetic drift) in promoting phenotypic and genetic divergence between the ecotypes, identifying the drivers of parallel evolution in New Zealand *Gobiomorphus*, and determining the extent to which the patterns observed here are found in other *G. cotidianus *populations.

## Methods

### Fish sampling and processing

Samples were collected during summer (Jan-Feb 2004 and Jan-Feb 2005) from Lake Tarawera, upstream Tarawera River and downstream Tarawera River (Figure 1). The upstream site is located at the edge of the fast-flowing upper river reaches, whereas the downstream site is located within a slower-flowing wetland area. The two sites are separated by paper-mill effluent discharges [52]. The Kaituna and the Rangitaiki River sites (also sampled in Jan-Feb 2004 and Jan-Feb 2005) were included to test for marine dispersal and to examine possible differences between river systems. Species were identified in the field and confirmed in the laboratory using meristic keys [34]. Captured *G. cotidianus *were transported to the laboratory, killed with MS-222 (tricaine methanesulfonate 0.1 g/L; Acros GmbH, Germany), weighed (± 0.001 g) and measured (total length ± 1.0 mm). For calculation of gonadosomatic indices, freshly dissected gonads of females were weighed (± 0.001 g). For meristic and otolith isotope analyses, samples were stored at -20°C. For genetic analyses, 50–150 μL of fresh blood was added to 50 μL of 0.05 M EDTA solution and immediately frozen at -80°C.

### Identification of canal morphotypes

Meristic analyses were conducted under a dissecting microscope on the supraorbital section of the oculoscapular canals of 112 fish (Table 1). Presence of canals was evident by pores at their extremities. The neuromasts anterior to the lateral canals shown in Figure 2 are replacement neuromasts (primary neuromasts; pn) that originated from canal neuromasts [81]. For data analyses, we defined canal types referring to these pore openings (Figure 2). Type 1 is characterized by paired median pores as well as anterior and posterior lateral pores. Type 2 has reduced median pores, hence only anterior and posterior lateral pores are present. Type 3 lacks any oculoscapular canals, hence it exhibits no pores. Canal types were recorded individually and proportions of canal types in sample locations were calculated (Table 2). To test for differences in canal morphotype proportions among sample sites, each fish was assigned a numerical identity for its morphotype and the Kruskal-Wallis ANOVA by ranks (*P *< 0.05) was used. Multiple pair-wise *post hoc *comparisons of mean ranks (two-tailed, *P *< 0.05) were conducted for all sites according to Siegel and Castellan [82] with Bonferroni correction.

### Otolith isotope analyses

Otoliths are composed of aragonitic calcium carbonate that is deposited continuously in concentric layers around a central nucleus. The incorporated amount of trace elements is proportional to the ambient environmental concentration [83,84]. Hence, otolith layers deposited during marine or estuarine residence exhibit higher ^88^Sr/^43^Ca ratios [85]. Consequently, these ratios are elevated in the nucleus of amphidromous individuals [83], including *G. cotidianus *[35]. Conversely, non-migratory individuals show constant, low ^88^Sr/^43^Ca ratios. Therefore, these relative differences between migratory and non-migratory individuals can be used to discriminate both ecotypes [35,86]. Isotope analyses were conducted on the left sagittal otolith of 60 individuals also included in the meristic analyses (Figure 1; Table 2). All diadromous *G. cotidianus *were identified in reference to two obligatorily diadromous *G. gobioides *individuals sampled from the Tarawera River. For otolith removal, the brain case was opened along the dorsal midline. Otoliths were removed, cleaned in distilled water and air dried for 24 h at room temperature before mounting, sanding and polishing. Otoliths were positioned horizontally on a microscope slide and embedded in thermosetting glue (Crystalbond, Aremco Products, Inc, USA). Upon curing, otoliths were sanded to the nucleus with a series of wetted carborundum papers (1200–4000 grit grades).

Transect readouts of ^88^Sr, ^137^Ba and ^43^Ca concentrations were carried out in a Perkin Elmer Elan SCIEX DRCII inductively coupled mass spectrometer with a New Wave Research Nd:YAG 213 nm wave length laser at the University of Waikato's Mass Spectroscopy Suite. Laser spot size was 30 μm, with a repetition rate of 20 Hz. A transect of line spots starting from the central nucleus to the edge was performed. Spacing between spots varied between 100–200 μm depending on otolith size. Laser power was set at 50% output with a five second firing and a ten second intersite pause between spots to allow dissipation of background analytes. Between samples the ablation chamber was purged for 90 s with the argon carrier gas. Additionally, the laser was fired at 0% power to standardize against interferences from the carrier gas. Control measurements were subtracted from sample counts per second (cps) to overcome any polyatomic interference. Isotope ratios were calculated from peak-cps for each otolith. Results are presented as dimensionless units for each isotope standardised to counts of ^43^Ca, an accepted technique in the absence of matrix-matched standards [87]. Results were expressed as line graphs, with each datum point representing the isotope ratio at that point of the otolith. Hence, each line represents the isotope profile across the otolith (Figure 3). Nucleus ^88^Sr/^43^Ca ratios against range of ^88^Sr/^43^Ca ratios of each individual profile were plotted to compare variation between individuals (Figure 4).

### Identification of spawning types

The fish included in spawning analyses were sampled during the peak time of summer spawning of *G. cotidianus *in that region (Jan-Feb 2004 and Jan-Feb 2005). *G. cotidianus *exhibits gonad development (indicated by gonadosomatic indices (GSI) > 1.0 and homogenous translucent ovaries) well in advance of spawning (3 to 5 months). Gonad development peaks during spawning and the gonadosomatic index drops significantly (mostly < 1.0) post spawning. Hence, independent of the season, the spawning status of *G. cotidianus *can be reliably determined by calculation of gonadosomatic indices that are complemented with direct observation of gonad development. Therefore, we defined different spawning types by calculation of gonadosomatic indices (gonad weight/(fish mass - organ mass) × 100)) for all females also included in the meristic analyses. Extensive fieldwork over several years has clearly identified the Rangitaiki River population as exclusively spawning in winter [52], permitting the use of their GSI data as references to define a threshold for winter spawners in our populations. As the highest GSI observed in the Rangitaiki River samples was 1.35, we assumed that all fish with a GSI below 1.35 were clearly not going to spawn during summer and were classified as winter spawners. With a GSI > 1.35, fish were developing gonads for spawning and were hence classified as summer spawners. All calculations were complemented by direct observations of gonadal development. The proportion of each spawning type in each sample site was calculated and plotted (Table 1; Figure 6). Statistical comparisons of spawning type proportions between sample sites were conducted as described for the canal morphotypes.

### Amplified Fragment Length Polymorphisms

The genetic structure was inferred with Amplified Fragment Length Polymorphisms (AFLPs; [56]), a high resolution marker capable of resolving intraspecific differences such as those expected among different ecotypes. We used three selective primer combinations to generate fingerprints for 55 out of the 109 individuals included in the meristic analyses (Table 1). AFLPs were generated as described elsewhere [52]. To ensure reproducibility, all fingerprints were duplicated for two of the three selective primer combinations and no significant difference was found between duplicates (*F*_ST _= 0; *P *> 0.99; calculated as described below). Additionally, a sample of fingerprints was scored both with automated and manual fragment scoring and no significant differences were found between methods, allowing automated scoring to be used for all data. Fragments in the size range of 50–500 bp were scored automatically in GENEMAPPER v3.7 [88] under default settings (peak height threshold = 100 rfu; bin width = 1.0 bp).

For each population, the percentage of polymorphic loci and the Shannon-Wiener diversity index (H_SH _= -∑ (p_j _ln p_j'_); where p_j _is the frequency of the j-th fragment) are given (Table 1). Fixation indices (*F*_ST_) [89] based on pair-wise distance between individuals (number of shared peaks in AFLP profiles) were calculated in ARLEQUIN v3.01 [90] with significance set to *P *< 0.05 (tested by 50,172 permutations among groups). Additionally, to detect population structuring the *F*_ST _analogue θ_B _was inferred using the software HICKORY [91]. HICKORY calculates θ_B _from dominant marker sets based on a Bayesian approach without having prior knowledge of population inbreeding [91]. Data collection was set to a burn-in of 50,000 iterations and data were collected for 250,000 runs. All HICKORY runs were duplicated to ensure repeatability and no significant differences were found between duplicates. Population dendrograms based on *F*_ST_, Reynolds' and Nei's genetic distances were generated in AFLPsurv v1. 0n [92] and consistency of clustering was tested by 1,000,000 bootstrapped distance matrices. A majority rule consensus tree was obtained from the bootstrapped distance matrices with the program routines NEIGHBOUR and CONSENSE from the PHYLIP v3.6 software package [93]. As all distance approaches were consistent we only present the dendrogram based on Nei's genetic distance (Figure 5A). Population structure was inferred with STRUCTURE v2.1 [94] utilizing the admixture model without prior population information. STRUCTURE determines population structure from multilocus genotype data based on a Bayesian clustering approach. During the analysis, STRUCTURE first assumes a number of populations ('clusters', K), then each individual is assigned to these populations, and, finally, for each K a posterior probability (lnP(D)) is given that describes the fit of the data to the respective K (for details about the simulation procedures see [94,95]). To infer the number of clusters K, STRUCTURE was implemented with a series of clusters (K = 1–7). Here, the burn-in was set to 100,000 generations and data were collected for 1,000,000 additional steps. For each K, five independent runs were performed to ensure reproducibility. As the most likely number of clusters present in a dataset is not necessarily indicated by the highest lnP(D) (see STRUCTURE manual for additional details) we applied the method of Evanno et al. [68] to approach the most likely number of clusters K. This method looks for a maximum of the slope (ΔK) of the lnP(D) distribution among runs.

### Comparison of the different analyses

To illustrate consistent patterns among the otolith, meristic, reproductive and genetic analyses we plotted the respective proportions (migratory type, morphotype, reproductive types and genotype) in each population and combined them in a single figure (Figure 6).

## Authors' contributions

MH, MS, and BJH conceived the study and CM wrote the first draft of the manuscript. MH collected the fish. CM and AC performed the AFLP fingerprinting and CM, BJH and RT collected the isotope and meristic data. CM, BJH, KS and MH performed the data analyses. All authors were involved in writing and data interpretation, and read and approved the final manuscript.
